# Evaluation of sparing organs at risk (OARs) in left‐breast irradiation in the supine and prone positions and with deep inspiration breath‐hold

**DOI:** 10.1002/acm2.12382

**Published:** 2018-06-21

**Authors:** Amitpal Singh Saini, Catherine S. Hwang, Matthew C. Biagioli, Indra J. Das

**Affiliations:** ^1^ Department of Medical Engineering University of South Florida Tampa FL USA; ^2^ Department of Radiation Oncology Florida Hospital Cancer Institute Orlando FL USA; ^3^ Department of Radiation Oncology NYU Langone Medical Center Laura and Isaac Perlmutter Cancer Center New York NY USA

**Keywords:** cardiac toxicity, deep inspiration breath‐hold, heart dose, LAD dose, left ventricle dose, Lt breast radiation, lung dose, prone position, supine position

## Abstract

**Purpose:**

To compare doses to organs at risk (OARs) for left‐sided whole‐breast radiation therapy with comparable planning target volume (PTV) coverage using three techniques: free breathing in a supine position (SFB), deep inspirational breath‐hold in a supine position (SDIBH), and free breathing in prone position (PFB).

**Materials and methods:**

Thirty‐three patients with left‐sided early‐stage breast cancer underwent CT simulation following SFB, SDIBH, and PFB protocols for whole‐breast radiation therapy. One radiation oncologist contoured the breast PTV, heart, left ventricle (LV), and left anterior descending artery (LAD). Treatment plans were optimized using field‐in‐field technique with the AAA algorithm. Each plan was optimized to provide identical coverage to the PTV such that a reasonable comparison for OAR dosimetry could be evaluated. All plans were prescribed 42.56 Gy in 16 fractions to the left‐breast PTV.

**Results:**

The mean dose in SFB for the heart, LV, and LAD was 1.92, 3.19, and 21.73 Gy, respectively, which were significantly higher than the mean dose in SDIBH for the heart (1.08 Gy, *P* ≤ 0.0001), LV (1.50 Gy, *P* ≤ 0.0001), and LAD (6.3 Gy, *P* ≤ 0.0001) and in PFB for the heart (0.98 Gy, *P* ≤ 0.0001), LV (1.34 Gy, *P* ≤ 0.0001), and LAD (6.57 Gy, *P* ≤ 0.0001). Similar findings were noted for the cardiac components in SFB for V2.5, V5, V10, V20, and V30 compared with values in SDIBH and PFB. The mean dose for the left lung in PFB was 0.61 Gy that was significantly lower than in SFB (5.63 Gy, *P *≤ 0.0001) and SDIBH (5.54 Gy, *P* ≤ 0.0001). Mean dose and dosimetric values for each OAR increased in SFB and SDIBH for patients with a large breast volume compared with values for patients with a small breast volume.

**Conclusions:**

SFB results in higher heart, LAD, and LV doses than the other techniques. Both PFB and SDIBH are more advantageous for these OARs irrespective of breast volume. PFB results in significantly lower lung doses than SFB and SDIBH. PFB always provided better results than SFB for the heart, LV, LAD, and lung. This conclusion contrasts with some published studies concluding that the prone position has no benefit for heart sparing.

## 1 INTRODUCTION

Lumpectomy followed by whole‐breast radiation therapy is considered the standard of care for the treatment of early‐stage breast cancer.^1^ While radiation therapy reduces the risk of local recurrence by 26% at 5 yr and improves overall survival by 5% at 15 yr, it is also associated with increased toxicity.^2^

Treatment of left‐sided breast cancers, in particular, results in increased risks of cardiac diseases and ischemic heart events,^2^, ^3^, ^4^, ^5^ and radiation doses delivered to the heart, left anterior descending artery (LAD), and lungs when patients are in a supine position remain significant.^6^, ^7^, ^8^ Darby et al.^2^ reported that an increase of 1 Gy to the mean dose to the heart results in a 7.4% relative increase in the risks of major coronary events. Another study demonstrated a significant increase in nonbreast‐cancer‐related mortality from heart disease with relative risk (RR, 1.27) and lung cancer (RR, 1.78) associated with breast radiation.^2^ However, these results are based on data using older radiation techniques and treatment modalities.

Modern radiation techniques, such as 3‐dimensional conformal radiation therapy (3DCRT), intensity‐modulated radiation therapy (IMRT), and volumetric‐modulated arc therapy (VMAT), are considered to decrease cardiac and pulmonary doses, while providing excellent coverage to the target volume with proper optimization.^5^, ^9^, ^10^, ^11^

With advances in cancer diagnosis and management techniques, patients are diagnosed early and live longer and are, therefore, at increased risk of developing long‐term complications from the treatment. Different techniques are used to reduce doses to organs at risk (OARs) without compromising coverage of the target volume. Deep inspiration breath‐hold (DIBH) is one such technique that has been shown to reduce cardiac doses.^12^, ^13^ Breast radiation while in a prone position is another technique that is utilized to minimize the dose to the heart and underlying lung.^14^, ^15^, ^16^, ^17^ However, no consensus has been reached in terms of the best treatment strategy between techniques utilizing free breathing in a supine position and in a prone position.^13^, ^16^, ^18^, ^19^, ^20^, ^21^, ^22^, ^23^

The aim of this study is to compare dosimetric parameters of various OARs in three different treatment positions for the same patient during left‐sided whole‐breast radiation therapy: a standard free‐breathing supine position (SFB), a supine position with a deep inspiration breath‐hold (SDIBH), and a free‐breathing prone position (PFB). In addition, dosimetric parameters were also evaluated and compared for three positions with respect to the breast volume of the patients.

## 2 MATERIALS AND METHODS

Between August 2015 and July 2016, 33 patients underwent whole‐breast radiation therapy for early‐stage left‐breast cancer (pathologic T1‐2N0 disease) were included in this retrospective study with approval from the institutional review board (IRB). Eligibility was not restricted based on the size or volume of the breast or the whole‐breast planning target volume (PTV). Only those patients who could follow instructions and hold their breath for a minimum of 25 s were considered suitable to be included in this study. A Vac‐Lock positioning cushion was used to immobilize patients in the supine position. A Bionix prone‐positioning breast board and Vac‐Lock cushion were used to immobilize patients in the prone position. Prior to scanning, the radiation oncologist marked the borders of the breast with a radio‐opaque wire. All scans were performed with a GE light speed RT scanner, model no 2266521.

**Figure 1:**
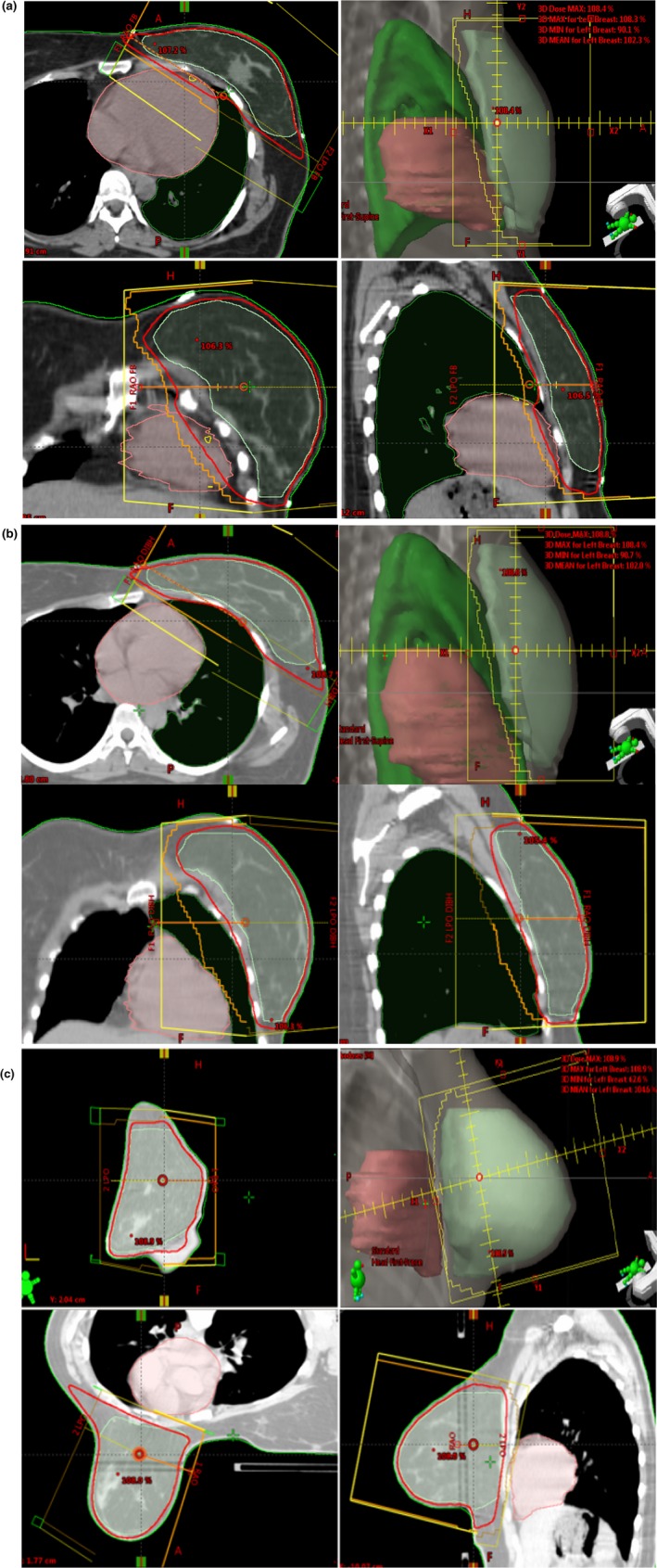
Clockwise, axial, beam's eye view, sagittal, and coronal images. Green line indicates PTV, red indicates 95% iodose line, orange indicates MLC field shape, and yellow indicates beam outline. Heart and lung volumes are also shown. (a) Supine free breathing (SFB), (b) supine deep inspirational breath‐hold (SDIBH) and (c) prone‐free breathing (PFB). Note the heart and lung positions in three techniques with respect to beam geometry.

The patients were first CT scanned in the SFB position, the second CT scan was done according to the SDIBH protocol established in our institution, and the third CT scan was done following the PFB protocol. Our SDIBH protocol entailed marking the patient in the medial and lateral directions with respect to CT lasers while breathing freely. Patients were then coached to take deep breath and hold it. New positions in the medial and lateral directions were marked on the patient's skin. Patients were again asked to take deep breath so that the CT simulation therapist could verify the consistency of the breath with respect to the lasers. Audio coaching was used to guide the patients through the breath‐holding process. High‐defini tion cameras were installed in the treatment room to clearly observe the marks made during the SDIBH procedure from outside. These cameras were also used to check the position of the patients during treatment with respect to the lasers. Table 1 presents the SDIBH simulation data sheet used during CT simulation to record positional shifts with respect to laser marks during the SFB protocol. After CT simulation following the SDIBH protocol, a third CT scan was taken following the PFB protocol. Figs. 1(a)–1(c) shows typical scans and beam placement on a patient indicating anatomy and locations in various techniques.

**Table 1:** Table for recording SDIBH measurements

Voluntary breath‐hold details
Max breath‐hold achieved (sec)
Anterior FB bb and BH bb, distance (mm)
Lateral FB bb and BH bb, distance (mm)

After the CT scans were obtained, images were transferred to the treatment planning system (TPS). At our institute (Florida Hospital Cancer Center), we use Eclipse TPS (Varian Medical System, version 11, Palo Alto, CA, USA). One radiation oncologist who specializes in breast contoured the breast PTV, heart, LV, LAD, and contralateral breast of each patient using the RTOG‐1304^24^ guidelines and RTOG Breast Cancer Atlas for planning (https://www.rtog.org/CoreLab/ContouringAtlases/BreastCancerAtlas.aspx). According to the atlas, breast was defined as all apparent CT glandular breast tissue, while taking into account the RTOG consensus definition of anatomical borders. Cranial border was defined at the second rib insertion. Caudal border was defined as the loss of CT apparent breast tissue. Anterior boundary was defined as the skin. Posterior boundary was the anterior aspect of the pectoralis muscles. Medial border was the sternal‐rib junction and lateral border was at the mid‐axillary line. The LAD was defined as the vessel that descended anteriolaterally from the anterior interventricular groove down to the apex of the heart.^25^ Cardiac contouring started superior at the level of the great vessel insertion into the heart and extended inferior to the apex of the heart. Contours were drawn by one physician for consistency. The lungs were contoured using an automatic segmentation tool available in Eclipse TPS, and lung contours were manually edited by physician as needed. Breast PTVs were cropped 5 mm from the skin surface for planning purposes as dosimetry in the buildup region is not well defined.^26^ The contralateral breast was not cropped from the skin surface.

Treatments for all patients were planned with a field‐in‐field (FIF) tangential beam technique, and no wedges were used in any plan. Only a 6‐MV beam was used for all three techniques. Treatments for all patients were planned using a hypofractionated fractionation scheme as defined by Whelan et al.^27^ Doses were prescribed as a total dose of 42.56 Gy in 16 fractions. Dose calculations were performed using the Anisotropic Analytical Algorithm (AAA Version 11.0.31) with a grid size of 0.25 × 0.25 cm^2^. Treatment plans were normalized to an isocenter placed in the PTV. As per the RTOG‐1304 protocol, a 7‐mm margin was added to the PTV to form the field shapes using MLC.^24^ All plans were optimized according to specified constraints to ensure that the data were comparable, and 95% of the PTV was prescribed to receive 100% of the prescribed dose while achieving maximum sparing of OARs. Dose‐volume histograms were used to analyze the dosimetry in PTV, dose homogeneity, and doses to OARs. Dosimetric values for the mean dose, V2.5, V5, V10, V20 and V30, were recorded and evaluated for all OARs. In addition, dosimetric parameters were also evaluated within each technique with respect to a small and large breast volume.

### 2.A Statistical analysis

The mean dose, V2.5, V5, V10, V20, and V30, was compared between SFB and SDIBH, SFB and PFB, and SDIBH and PFB plans. All the dosimetry parameters for the heart, LV, LAD, and left lung were determined using a Wilcoxon signed‐rank test for related sample with SPSS statistical software, version 23.0, as data had a non‐normal distribution. Data were considered statistically significant at a *P* value ≤ 0.05.

## 3 RESULTS

### 3.A Volume analysis

Heart volume was smallest in PFB and largest in SFB. Mean heart volume was 592.4 cm^3^ (range, 380–967 cm^3^), 554 cm^3^ (range, 370–712 cm^3^), and 544 cm^3^ (range, 354–756 cm^3^) for SFB, SDIBH, and PFB, respectively. Whole‐breast PTV volumes ranged between 330 and 1723 cm^3^. Mean whole‐breast PTV volume was 654.2 cm^3^ (range, 299.8–1641 cm^3^), 660.6 cm^3^ (range, 253.2–1650.1 cm^3^), and 685.9 cm^3^ (range, 302–1723 cm^3^) for SFB, SDIBH, and PFB, respectively. Thus, mean whole‐breast PTV volume was highest in PFB and lowest in SFB.

Based on a literature search, a breast volume of 750 cm^3^ was chosen to divide the patients into two groups: patients with a small breast volume and patients with a large breast volume in this study.^22^, ^23^ Patients with a breast PTV < 750 cm^3^ were considered to have small breasts, and patients with a breast PTV ≥ 750 cm^3^ were considered to have large breasts. From the 33 patients evaluated in this study, 21 were considered to have small breasts, and 12 patients were considered to have large breasts.

### 3.B Dosimetric analysis

**Figure 2:**
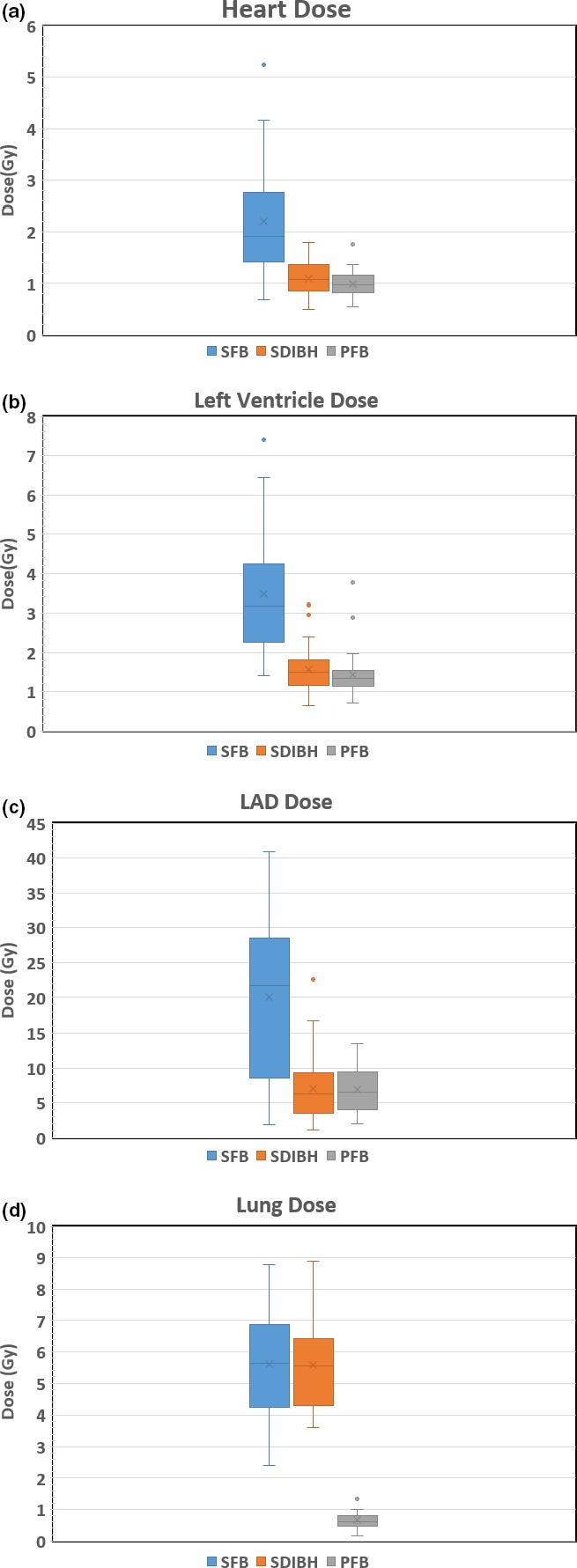
Box‐whisker plot of dose with error bars in supine free breathing (SFB), supine deep inspiration breath‐hold (SDIBH), and prone free‐breathing (PFB) techniques. Outlier data are shown if they existed for (a) heart, (b) left ventricle (LV), (c) left anterior descending artery (LAD), and (d) lung.

The mean dose to the heart was reduced by 50% in SDIBH and PFB as compared with the dose in SFB. The mean dose to heart in SFB was 1.92 Gy, compared with 1.11 Gy in SDIBH and 0.98 Gy in PFB, as shown in Fig. 2(a). Statistically significant differences were found for mean doses to the heart between SFB and SDIBH (*P* ≤ 0.0001) and between SFB and PFB (*P* ≤ 0.0001); however, no statistically significant difference was found between SDIBH and PFB (*P* = 0.114). Out of 33 patients, only one patient has higher mean heart dose in PFB as compared with SFB. All other dosimetric values were higher in SFB than in SDIBH and PFB, as shown in Table 2(a).

**Table 2:** Dosimetry parameters (median values and quartiles) in supine free breathing (SFB), supine deep inspiration breath‐hold (SDIBH), and prone free‐breathing (PFB) techniques. (a) Heart, (b) left ventricle (LV), (c) left anterior descending artery (LAD), and (d) left lung

	SFB	SDIBH	PFB
(a) Heart dose (Gy)			
Mean dose	1.92 (1.42–2.76)	1.08 (0.84–1.36)	0.98 (0.83–1.15)
V2.5	14.60 (9.27–22.34)	7.20 (3.98–11.46)	6.30 (4.47–8.89)
V5	4.81 (2.73–7.35)	0.90 (0.22–1.92)	0.80 (0.275–1.61)
V10	2.66 (1.43–4.58)	0.11 (0.00–0.86)	0.10 (0.01–0.47)
V20	1.74 (0.79–3.21)	0.00 (0.00–0.20)	0.00 (0.00–0.12)
V30	1.15 (0.23–2.34)	0.00 (0.00–0.17)	0.00 (0.00–0.00)
(b) Left ventricle dose (Gy)			
Mean dose	3.19 (2.25–4.24)	1.50 (1.15–1.80)	1.34 (1.13–1.54)
V2.5	30.00 (18.84–39.00)	11.54 (7.46–19.08)	8.92 (6.01–12)
V5	9.23 (5.24–14.23)	1.05 (0.38–3.29)	0.92 (0.43–1.88)
V10	5.01 (2.34–9.65)	0.00 (0.00–1.05)	0.30 (0.00–0.55)
V20	3.10 (1.29–6.27)	0.00 (0.00–0.19)	0.00 (0.00–0.15)
V30	2.04 (0.27–4.61)	0.00 (0.00–0.00)	0.00 (0.00–0.00)
(c) LAD dose (Gy)			
Mean dose	21.73 (8.55–28.5)	6.30 (3.51–9.31)	6.57 (3.99–9.49)
V2.5	95.70 (85.32–99.37)	84.62 (68.90–90.65)	87.50 (74.83–93.93)
V5	74.91 (55.86–93.16)	39.52 (11.12–61.87)	54.46 (27.45–66.70)
V10	61.50 (27.79–81.50)	12.66 (0.03–40.45)	19.50 (4.28–35.25)
V20	48.90 (13.30–73.27)	0.00 (0.00–9.02)	0.96 (0.00–9.49)
V30	36.33 (0.91–58.80)	0.00 (0.00–0.19)	0.00 (0.00–0.23)
(d) Left lung dose (Gy)			
Mean dose	5.63 (4.23–6.86)	5.54 (4.29–6.42)	0.61 (0.47–0.80)
V2.5	30.60 (25.75–38.3)	34.90 (28.04–39.21)	2.52 (1.85–4.49)
V5	19.99 (15.97–25.00)	21.23 (16.30–25.25)	0.95 (0.34–1.61)
V10	13.27 (9.76–17.16)	13.14 (9.84–16.37)	0.38 (0.05–0.865)
V20	9.84 (6.39–12.98)	9.34 (6.79–11.73)	0.10 (0.0–0.32)
V30	7.54 (4.78–10.26)	7.15 (4.81–8.79)	0.01 (0.0–0.13)

Mean LV dose was reduced by 47% in SDIBH and PFB compared with the dose in SFB. The LV received the largest mean dose, 3.19 Gy in SFB and received doses of 1.5 Gy in SDIBH and 1.34 Gy in PFB, as shown in Fig. 2(b). The mean dose to the LV was significantly reduced between SFB and SDIBH (*P* ≤ 0.0001) and between SFB and PFB (*P* ≤ 0.0001), but no statistically significant difference was found between SDIBH and PFB (*P* = 0.137). Out of 33 patients, only one patient has higher mean LV dose in PFB as compared with SFB. The LV dosimetry values were also found to be higher in SFB than in SDIBH and PFB. A marginally lower dosimetric values in PFB was observed compared with SDIBH, as shown in Table 2(b).

The mean LAD dose was highest, 21.73 Gy, in SFB and was 6.30 Gy in SDIBH and 6.57 Gy in PFB, as shown in Fig. 2(c). SDIBH and PFB resulted in a 70% reduction in the mean LAD dose compared with the dose in SFB. The mean dose for LAD was significantly reduced between SFB and SDIBH (*P* ≤ 0.0001) and between SFB and PFB (*P* ≤ 0.0001), but no statistically significant reduction in dose was found between SDIBH and PFB (*P* = 0.122). Out of 33 patients, six patients had higher mean LAD dose in PFB as compared with SFB. The LAD dosimetric parameters were also higher in SFB than in SDIBH and PFB, as shown in Table 2(c).

The mean dose to the lung was reduced by 89% in PFB compared with doses in SFB and SDIBH. The lung received 5.63 Gy in SFB, 5.54 Gy in SDIBH, and 0.61 Gy in PFB, as shown in Fig. 2(d). Differences in mean doses to the lung were not statistically significant between SFB and SDIBH (*P* = 0.964), but doses were significantly different between SFB and PFB (*P* ≤ 0.0001) and between SDIBH and PFB (*P *≤ 0.0001). All other dosimetric values for the lung were also the lowest in PFB, as shown in Table 2(d).

The P values were also calculated for all the OARs and for all dosimetric parameters to identify statistically significance differences between the techniques, as shown in Table 3. SDIBH and PFB were significantly better than SFB according to all the dosimetric parameters for the heart, LV, and LAD, but there was no significant difference between SDIBH and PFB, except in V5 for the LAD. The left lung was significantly less at risk in PFB than in SFB and SDIBH for all the dosimetric parameters evaluated in this study.

**Table 3:** *P* value between PFB and SDIBH, PFB and SFB, PFB and SDIBH for all the dosimetric parameters of heart, LV, LAD, and lung. Please note that *P* values for heart, LV, LAD are statistically significant between SDIBH and SFB, and PFB and SFB. *P* values ≤ 0.05 were considered statistically significant

Dose/volume	Technique	Heart	LV	LAD	Lung
Mean	SDIBH and SFB	0.000	0.000	0.000	0.964
	PFB and SFB	0.000	0.000	0.000	0.000
	PFB and SDIBH	0.114	0.137	0.122	0.000
V2.5	SDIBH and SFB	0.000	0.000	0.000	0.0080
	PFB and SFB	0.000	0.000	0.034	0.0000
	PFB and SDIBH	0.242	0.055	0.211	0.0000
V5	SDIBH and SFB	0.000	0.000	0.000	0.0560
	PFB and SFB	0.000	0.000	0.000	0.0000
	PFB and SDIBH	0.936	0.335	0.007	0.0000
V10	SDIBH and SFB	0.000	0.000	0.000	0.7791
	PFB and SFB	0.000	0.000	0.000	0.0000
	PFB and SDIBH	0.765	0.746	0.153	0.0000
V20	SDIBH and SFB	0.000	0.000	0.000	0.6739
	PFB and SFB	0.000	0.000	0.000	0.0000
	PFB and SDIBH	0.932	0.935	0.627	0.0000
V30	SDIBH and SFB	0.000	0.000	0.000	0.5143
	PFB and SFB	0.000	0.000	0.000	0.0000
	PFB and SDIBH	0.569	0.311	0.955	0.0000

### 3.C Dosimetric analysis based on breast volume

Mean doses to all OARs in patients based on breast PTV are shown in Table 4. Differences for all dosimetric parameters between all three techniques with respect to small and large breast volumes are shown in Table 5. Doses to the heart, LV, LAD, and lung in SFB and SDIBH are higher for large‐volume breasts than for small‐volume breasts. In contrast, in PFB, most of the dosimetric values for all of the OARs were lower for patients with large breasts.

**Table 4:** Dosimetric parameters (median values and quartiles) of OARs in SFB, SDIBH, and PFB based on breast PTV volume <750 cm^3^ and >=750 cm^3^. Please note than PFB has the lowest mean values for heart, LV, LAD, and lung for breast PTV volume >=750 cm^3^

Breast PTV volume <750 cm^3^	SFB	SDIBH	PFB
Mean heart dose (Gy)	1.65 (1.12–2.32)	0.87 (0.71–1.21)	0.90 (0.81–1.10)
Mean LV dose (Gy)	2.93 (1.85–4.04)	1.30 (1.01–1.70)	1.32 (1.13–1.50)
Mean LAD dose (Gy)	19.86 (7.85–25.1)	5.97 (3.01–8.53)	6.5 (3.58–9.16)
Mean lung dose (Gy)	5.48 (3.93–6.52)	5.06 (4.09–6.38)	0.61 (0.48–0.97)
Breast PTV volume >=750 cm^3^			
Mean heart dose (Gy)	2.59 (1.87–4.06)	1.36 (0.97–1.62)	1.07 (0.87–1.31)
Mean LV dose (Gy)	3.61 (3.02–5.77)	1.72 (1.40–2.11)	1.2 (1.11–1.58)
Mean LAD dose (Gy)	24.74 (10.22–36.75)	7.05 (3.27–12.99)	6.7 (4.51–9.93)
Mean lung dose (Gy)	5.69 (4.77–7.08)	5.7 (5.23–7.06)	0.57 (0.36–0.68)

**Table 5:** Dosimetric differences of median values between each technique, that is, SFB‐SDIBH, SFB‐PFB, SDIBH‐PFB, based on breast PTV volume

Breast PTV volume <750 cm^3^	SFB‐SDIBH	SFB‐PFB	SDIBH‐PFB
Mean heart dose (Gy)	0.78	0.75	−0.03
Mean LV dose (Gy)	1.63	1.61	−0.02
Mean LAD dose (Gy)	13.96	13.36	−0.6
Mean lung dose (Gy)	0.42	4.87	4.45
Breast PTV volume >=750 cm^3^			
Mean heart dose (Gy)	1.235	1.525	0.29
Mean LV dose (Gy)	1.89	2.415	0.525
Mean LAD dose (Gy)	17.695	18.045	0.35
Mean lung dose (Gy)	−0.005	5.12	5.125

## 4 DISCUSSION

Radiation‐induced cardiac toxicity and injury after radiation therapy treatment for left‐sided breast cancers are well documented in the literature.^2^, ^3^, ^4^, ^5^, ^28^ The rate of major coronary events increases linearly with mean radiation doses to the heart without any threshold.^3^, ^5^, ^28^ Thus, it is important to find treatment techniques that will lower the dose to cardiac components without compromising the target coverage.

Das et al.^29^ provided an analytical approach correlating lung and heart doses to pulmonary and cardiac complication rates. Therefore, it is also important to reduce doses to OARs such as the left lung and contralateral breast to reduce the risk of pneumonitis, lung fibrosis, and secondary cancers, especially in patients who are expected to have long‐life expectancies.^2^, ^3^, ^4^

The PFB uses gravity to pull the treated breast away from the heart and lung, thus resulting in dose reduction to OARs. Also, in PFB, with careful planning, one can minimize the treatment fields going through the heart without compromising PTV coverage. A literature search yielded mixed results on the benefits of PFB for heart sparing. Some studies have reported that the prone position reduces heart doses,^15^, ^23^ but other studies have concluded that this position is only beneficial for patients with a large breast volumes.^14^, ^16^, ^22^ It has been reported that in some patients, heart doses in the prone position increase because of the proximity of the heart to the treated area.^18^, ^20^ A few studies have indicated that PFB provides no benefits for sparing the heart.^21^, ^30^ Formenti et al.^23^ reported that the benefits of PFB are statistically significant compared with the results of SFB when breast volume is larger than 750 cm^3^.

Our results suggest that the mean heart dose can be reduced by almost half using SDIBH and PFB compared with using SFB. When the patient takes a deep breath, the heart moves posteriorly and inferiorly due to lung expansion and diaphragmatic movements. Thus, the heart moves away from the chest wall. Moving of heart during SDIBH helps in reducing the volume of the heart in the treatment field, reducing the dose to the heart. The mean dose and values for all the dosimetric parameters were lowest in PFB for the LV. It is believed that the dose to the LAD plays a vital role in radiation‐induced cardiac toxicity.^31^, ^32^, ^33^ The mean dose to the LAD was found to be similar for SDIBH and PFB, and highest mean dose was in SFB. In a similar study, Venhoven et al.^30^ concluded that PFB results in higher doses to the heart and LAD than the SFB and SDIBH techniques, but the results of our study are different as both SDIBH and PFB led to lower heart and LAD doses than SFB, irrespective of the breast volume. A significant reduction in V2.5, V5, V10, V20 and V30 for the heart, LV and LAD in SDIBH and PFB was observed compared with values in SFB.

We found equivocal results related to the reduction of radiation doses to the heart in PFB in the literature search. However, all studies agree that lung dose are dramatically reduced in PFB compared with doses in SFB and SDIBH.^14^, ^15^, ^16^, ^17^, ^21^, ^22^, ^23^, ^30^ Lung doses are significantly lower in PFB than in SFB and SDIBH. The lung density of the irradiated lung volume decreases also in SDIBH.^10^, ^15^, ^30^ One study mentioned that the opposite occurs in PFB, as the lungs are pushed downward by gravity and consequently lung density may increase.^30^ However, PFB showed clear advantages over SFB and SDIBH for lowering lung doses and the values of most other dosimetric parameters compared with SFB in this study.

We did not find any other study in literature search that has evaluated the heart, LV, LAD, and lung for V2.5, V5, V10, V20, V30 and statistically compared each dosimetric parameter between the techniques and that has also compared dosimetric differences in OARs for SFB, SDIBH, and PFB with respect to breast volume. Mean doses evaluated for each OAR increased in SFB and SDIBH going from patients with small to large breast volumes, as shown in Table 4. This is because as breast volume increases, the separation between fields also increases, thus irradiating a larger volume to cover the PTV adequately. A large breast volume also requires wider beams to cover it, thus radiating a larger volume in SFB and SDIBH and leading to higher doses to cardiac components and the lung.

An interesting observation is that differences in doses and in dosimetric parameters evaluated between SFB and SDIBH and between SFB and PFB increased from patient with small to large breast volumes, as shown in Table 5. Thus, SDIBH and PFB are even more beneficial than SFB for patients with large breasts.

## 5 CONCLUSION

It is concluded that radiation dose can be significantly reduced to the heart, LV, LAD, and lung with the selection of the proper technique. PFB is obviously preferred dosimetrically over SFB and SDIBH. PFB is more beneficial than SFB for OARs sparing irrespective of breast volumes. SDIBH and PFB deliver lower doses to cardiac components than SFB. PFB delivers significantly lower lung doses than SFB and SDIBH. Thus, PFB could be the treatment of choice for patients with underlying pulmonary diseases. In addition, a patient‐specific analysis, patient anatomy, patient comfort, selection of beam arrangements, and breathing patterns should be given consideration in the selection process of techniques to treat breast cancer.

## CONFLICT OF INTEREST

No conflict of interest to declare.
